# The motilin agonist erythromycin increases hunger by modulating homeostatic and hedonic brain circuits in healthy women: a randomized, placebo-controlled study

**DOI:** 10.1038/s41598-018-19444-5

**Published:** 2018-01-29

**Authors:** Dongxing Zhao, Anne Christin Meyer-Gerspach, Eveline Deloose, Julie Iven, Nathalie Weltens, Inge Depoortere, Owen O’daly, Jan Tack, Lukas Van Oudenhove

**Affiliations:** 1 Translational Research Center for Gastrointestinal Disorders, Catholic University of Leuven, Leuven, Belgium; 2 Department of Research, Clara Hospital Basel, Basel, Switzerland; 3 King’s college London’s Institute of Psychiatry, London, UK

## Abstract

The motilin agonist, erythromycin, induces gastric phase III of the migrating motor complex, which in turn generates hunger peaks. To identify the brain mechanisms underlying these orexigenic effects, 14 healthy women participated in a randomized, placebo-controlled crossover study. Functional magnetic resonance brain images were acquired for 50 minutes interprandially. Intravenous infusion of erythromycin (40 mg) or saline started 10 minutes after the start of scanning. Blood samples (for glucose and hormone levels) and hunger ratings were collected at fixed timepoints. Thirteen volunteers completed the study, without any adverse events. Brain regions involved in homeostatic and hedonic control of appetite and food intake responded to erythromycin, including pregenual anterior cingulate cortex, anterior insula cortex, orbitofrontal cortex, amygdala, caudate, pallidum and putamen bilaterally, right accumbens, hypothalamus, and midbrain. Octanoylated ghrelin levels decreased, whereas both glucose and insulin increased after erythromycin. Hunger were higher after erythromycin, and these differences covaried with the brain response in most of the abovementioned regions. The motilin agonist erythromycin increases hunger by modulating neurocircuitry related to homeostatic and hedonic control of appetite and feeding. These results confirm recent behavioural findings identifying motilin as a key orexigenic hormone in humans, and identify the brain mechanisms underlying its effect.

## Introduction

The bidirectional neural and hormonal communication system between the brain and the gastrointestinal (GI) tract is known as the ‘brain-gut axis’^1^. It is part of an integrated interoceptive system which continuously conveys homeostatic information about the physiological state of the body to the brain.

GI hormones are important mediators of these gut-brain interactions^1^. One of these hormones, motilin, is a 22-amino-acid gut peptide secreted by endocrine M cells in the small intestine^2^. Motilin is a physiological regulator of the migrating motor complex (MMC), a cyclical contraction pattern with different phases of activity (phase I-III) originating in the stomach or duodenum and migrating distally in the fasted state^3,4^. Phase III is characterized by strong contractile activity. In healthy humans, gastric but not duodenal phase IIIs are preceded by a motilin peak and exogenous administration of motilin induces a premature gastric phase III^5,6^, which has recently been associated with increases in hunger ratings and with the occurrence of hunger peaks during fasting^7^.

Although motilin has thus been associated with the induction of these so-called hunger contractions^8^, the brain mechanisms underlying the putative role of motilin in regulating appetite and eating behavior have not been studied until now. This is partially due to the limited availability of appropriate animal models to study its effect, as motilin and its receptors only exist as pseudogenes in rodents and are not functional^9^. Therefore, the gut-brain signaling mechanisms underlying the putative orexigenic effect of motilin can only be studied in humans and other non-rodent species. Furthermore, as intravenously administerable motilin is no longer available for human research, motilin receptor agonists need to be used to study its physiological effects in humans. Two decades ago, Itoh *et al*.^10^ first found that the macrolide antibiotic erythromycin mimics the effect of exogenous motilin on GI contractile activity in dogs. Later, this was also observed in humans^10,11^ and erythromycin was shown to be an agonist of motilin receptors in the human duodenum and colon^12,13^.

Recent studies by our group^4,8^ showed that i.v. infusion of a low dose (40 mg) of erythromycin induces gastric phase III contractions accompanied by peaks in hunger ratings, not only after prolonged fasting but also in the interprandial state (120 minutes after a 250 kcal meal). Moreover, subjects requested a meal more often after erythromycin administration compared to placebo^7^. Cholinergic blockade by atropine suppressed both gastric phase III contractions and hunger peaks associated wih erythromycin administration. Nevertheless, the mechanism behind the induction of hunger by motilin receptor stimulation is incompletely understood. However, it is conceivable that this orexigenic effect is due to changes in activity in brain circuits involved in the control of appetite and food intake through the gut-brain axis.

Our primary aim was therefore to identify the brain mechanisms underlying the orexigenic effects of motilin receptor activation through erythromycin infusion. We hypothesized that intravenous erythromycin administration would increase hunger ratings and hedonic food intake compared to placebo, and that these effects would be accompanied by changes in activity in homeostatic and hedonic brain regions. Further, it has been shown in animal models that the orexigenic GI hormone ghrelin is essential for motilin-induced gastric contractions^14^, and motilin (or its agonists) increase plasma insulin levels in both healthy humans and animals^15,16^. A second aim was therefore to explore the putative role of ghrelin and insulin in these effects.

## Results

### Study participants

Fourteen eligible volunteers with a mean age of 26 ± 2 years and mean BMI of 21.4 ± 0.5 kg/m^2^ were recruited (June 2015–February 2016). One volunteer was excluded from analysis after scanning because she concealed a medical history and current symptoms of functional dyspepsia. All analyses were performed on the remaining 13 volunteers (age: 26 ± 2 years, BMI: 21.2 ± 0.5 kg/m^2^). The characteristics of the participants are listed in Table 1. An overview of the recruitment procedure is presented in Supplementary Figure S1. Minimal nausea scores (zero-inflated with very limited variability between conditions, time points and subjects, not permitting formal statistical analysis) were reported. No other adverse events occurred.

**Table 1:** Characteristics of participants at baseline in the erythromycin and the saline (placebo) conditions.

	**Healthy volunteers**	
*n*	13	
*Age (years)*	26 ± 2	
*BMI (kg/m*^2^)	21.2 ± 0.5	
	**Erythromycin condition**	**Saline condition**
*Hunger (mm)*	74 ± 4	66 ± 5
*Prospective food consumption (mm)*	73 ± 4	68 ± 4
*Fullness (mm)*	5 ± 2	10 ± 3
*Satiety (mm)*	8 ± 2	21 ± 7
*Plasma octanoylated ghrelin (pg/mL)*	48 ± 7	36 ± 5
*Plasma motilin (pg/mL)*	732 ± 31	716 ± 32
*Blood glucose (mg/dl)*	92 ± 4	92 ± 3
*Plasma insulin (µU/mL)*	21 ± 5	15 ± 3

### Appetite-related sensations

#### Hunger and prospective food consumption

**Figure 1:**
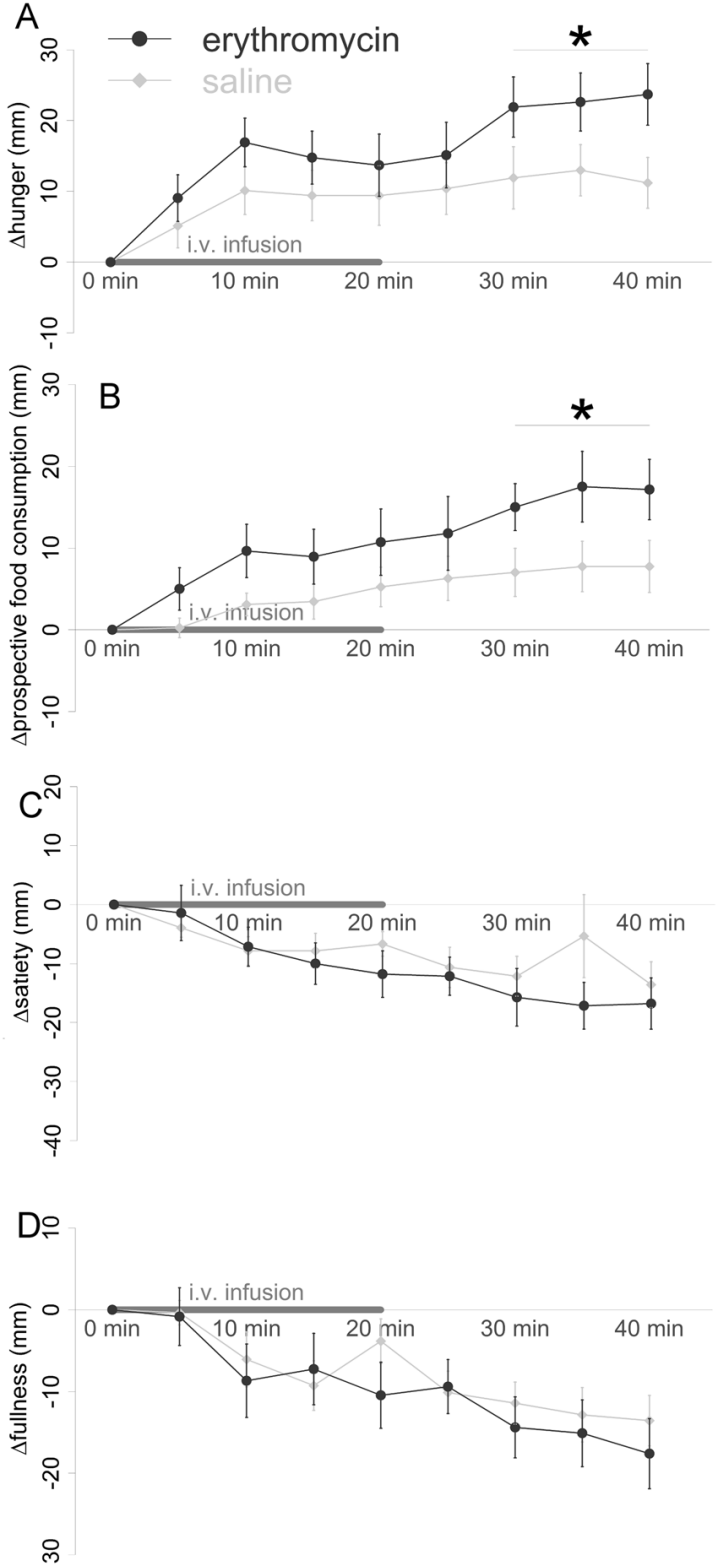
Appetite-related sensation changes after erythromycin infusion compared to saline. (**A**) The increase in hunger ratings was higher after erythromycin infusion in the time period t = 30–40 min (*p < 0.05). (**B**) The increase in prospective food consumption ratings was higher after erythromycin infusion in the time period t = 30–40 min (*p < 0.05). (**C**) The decrease in satiety ratings was not significantly different after erythromycin compared to saline infusion in the time period t = 30–40 min (p = 0.21). (**D**) The decrease in fullness ratings was not significantly different after erythromycin compared to saline infusion in the time period t = 30–40 min (p = 0.34). Data are shown as mean ± SEM.

As hypothesized, the increase in hunger and prospective food consumption in the time period t = 30–40 min was significantly higher after erythromycin compared to placebo (planned contrast, lower tailed t-test, p = 0.023, p = 0.022, respectively) (Fig. 1A and B).

#### Satiety and Fullness

The decrease in satiety and fullness in the time period t = 30–40 min did not differ between the two conditions (planned contrast, lower tailed t-test, p = 0.21, p = 0.34, respectively) (Fig. 1C and D).

Results of the full mixed model analyses are provided in Supplement.

### Hormone responses

#### Plasma octanoylated ghrelin

**Figure 2:**
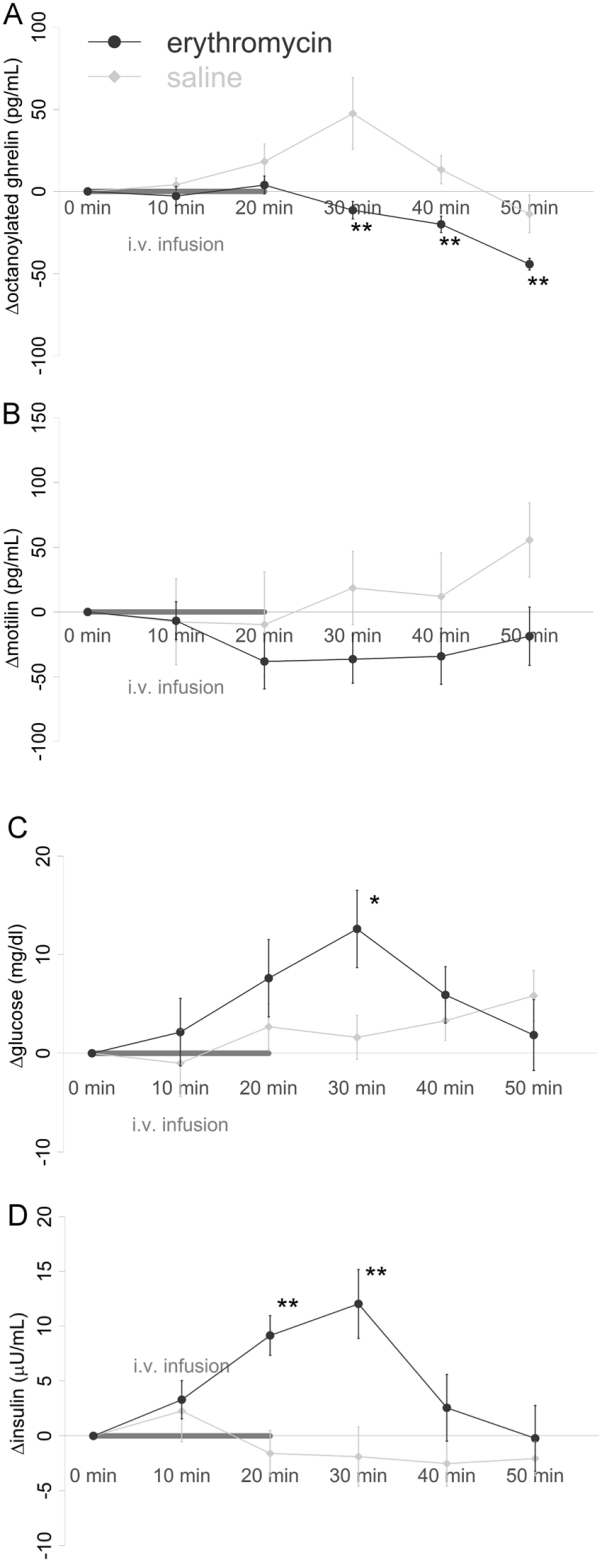
Plasma hormone and blood glucose changes after erythromycin infusion compared to saline. (**A**) Plasma octanoylated ghrelin levels decreased after erythromycin infusion, and increased after placebo infusion. The difference between erythromycin and placebo was significant at t = 30, 40, and 50 min (**p < 0.01). (**B**) Plasma motilin levels decreased after erythromycin infusion, and increased after placebo infusion, but this difference was not significant. (**C**) Blood glucose levels increased after erythromycin compared to placebo. The difference between erythromycin and placebo was significant at t = 30 (*p < 0.05). (**D**) Plasma insulin level increased after erythromycin infusion compared to placebo infusion. The difference between erythromycin and placebo was significant at t = 20 and 30 min (**p < 0.01). Data are shown as mean ± SEM.

A significant main effect of condition was found (F_1,23_ = 10.37, p = 0.004), with erythromycin inhibiting the increase in ghrelin found in the placebo condition. Further, the condition-by-time interaction was significant (F_4,96_ = 3.46, p = 0.011). *Post-hoc* t-tests indicated that this was driven by a significantly stronger decrease in ghrelin (relative to baseline) after erythromycin versus placebo at t = 30 min, 40 min, and 50 min, p_Holm_ = 0.003, 0.007, and 0.005 respectively (Fig. 2A).

#### Plasma motilin

No main effect of condition was found (F_1,107_ = 1.66 p = 0.20). Furthermore, no condition-by-time interaction was found (F_4,107_ = 1.11, p = 0.36) (Fig. 2B).

#### Blood glucose

No main effect of condition was found (F_1,12_ = 1.52, p = 0.24). However, a significant condition-by-time interaction effect was found (F_4,48_ = 2.89, p = 0.032). *Post-hoc* t-tests indicated a significantly higher increase of blood glucose level at t = 30 min, p_Holm_ = 0.043 (Fig. 2C).

#### Plasma insulin

A significant main effect of condition was found (F_1,107_ = 8.36 p = 0.005), with erythromycin increasing plasma insulin compared to placebo over the entire period of scanning. Further, the condition-by-time interaction was significant (F_4,107_ = 6.9, p < 0.0001). *Post-hoc* t-tests indicated that this was driven by a significantly stronger increase in insulin (relative to baseline) after erythromycin versus placebo at t = 20 min and 30 min, p_Holm_ = 0.0005 and 0.0024 respectively) (Fig. 2D).

### Hedonic food intake

Subjects tended to drink more milkshake after erythromycin compared to placebo infusion (449 ± 41 g vs. 391 ± 40 g, upper-tailed paired t-test, t12 = 1.6, p = 0.068, Cohen’s d: 0.44).

### Pharmacological megnatic resonance imaging (phMRI)

**Figure 3:**
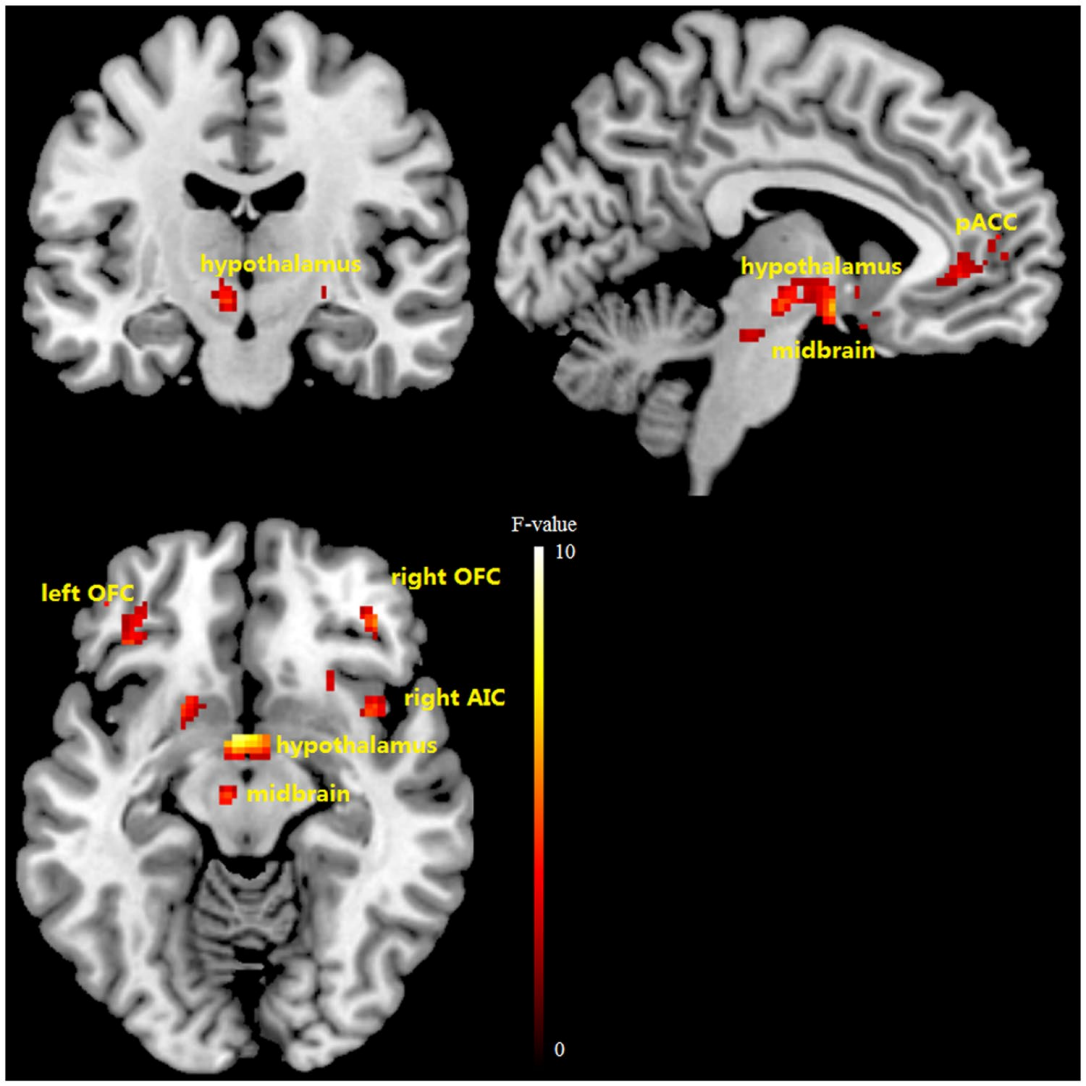
Differential brain response to erythromycin infusion versus saline in a mask of pre-hypothesized regions of interest. Color bar represents F-values. pACC, perigenual anterior cingulate cortex; AIC, anterior insular cortex; OFC, orbitofrontal cortex.

**Figure 4:**
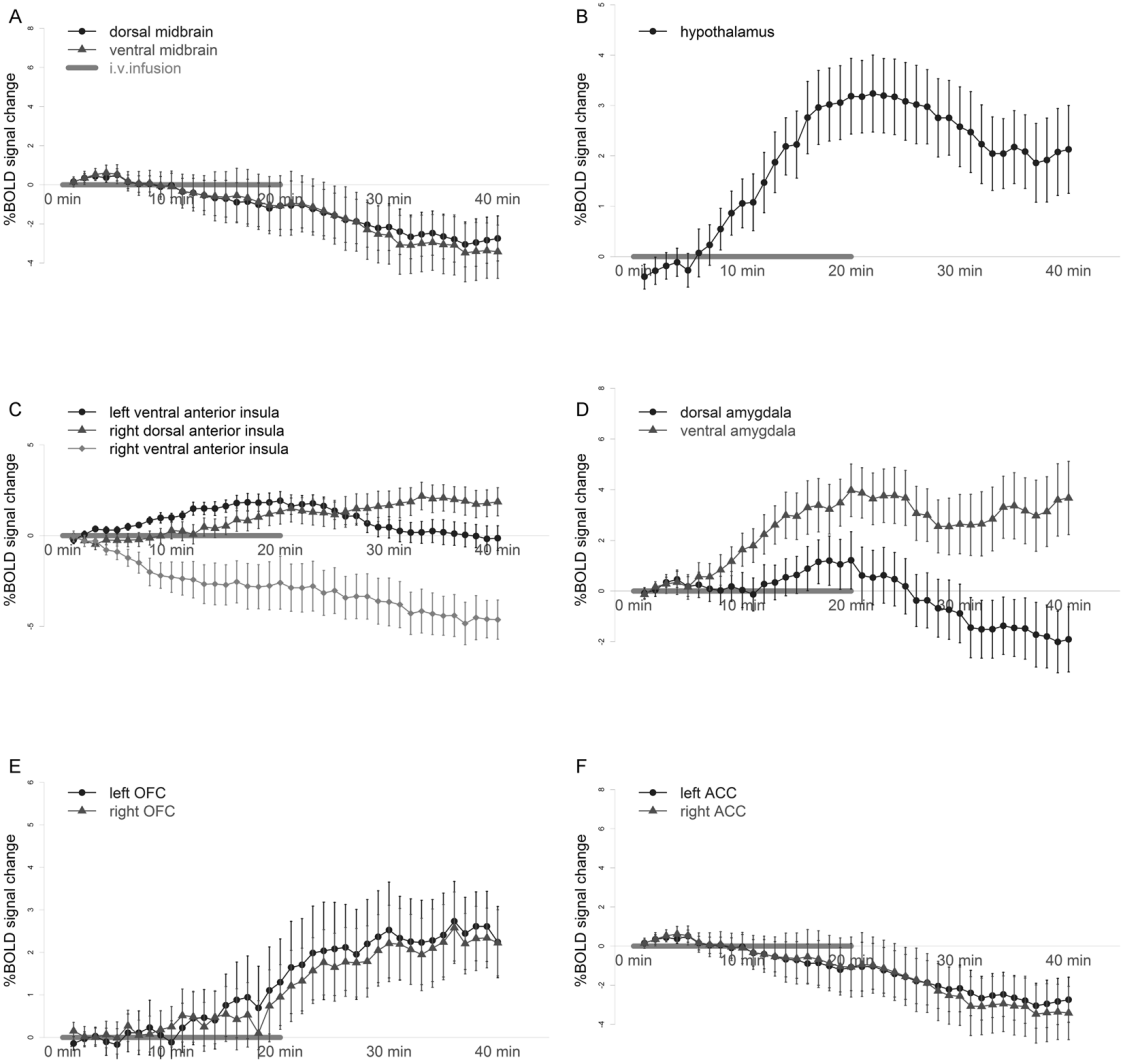
Blood oxygen level dependent (BOLD) signal change percentage differences between erythromycin and saline infusion in representative brain regions. (**A**) Dorsal and ventral midbrain, (**B**) hypothalamus, (**C**) bilateral anterior insula, (**D**) bilateral amygdala, (**E**) bilateral orbital frontal cortex (OFC), (**F**) bilateral anterior cingulate cortex (ACC). Data are shown as mean ± SEM.

A significant condition-by-time interaction effect on the blood oxygen level dependent (BOLD) signal was found in pre-hypothesized region of interests (ROIs) including pregenual anterior cingulate cortex (pACC), anterior insula cortex (AIC), orbital frontal cortex (OFC), amygdala, caudate, pallidum and putamen bilaterally, right nucleus accumbens, hypothalamus, and midbrain. Numerical details are shown in Table 2, results are visually represented in Fig. 3 and the time course of the difference in response in selected ROIs is plotted in Fig. 4. Some of these regions showed an increase in activity in response to motilin versus saline, while others showed a decrease.

**Table 2:** Differential brain response to erythromycin infusion versus saline in a mask of pre-hypothesized regions of interest.

**Region**	**Side**	**Activity**	**x (mm)**	**y (mm)**	**z (mm)**	**Cluster volume**	**pFWE-corrected**	**F-peak**
*Anterior insula cortex*	Left	↑	−24	14	−15	130	<0.0001	7.6
	Right	↑/↓	38	12	−13	39	0.0004	5.7
*Nucleus Accumbens*	Right	↑	6	8	−11	1	0.03	2.5
*Amygdala*	Left	↑/↓	−30	−4	−23	12	0.005	4.0
	Right	↑	28	−6	−13	41	0.0003	3.9
*Caudate*	Left	↓	−12	6	23	2	0.025	2.6
	Right	↑	14	12	15	35	0.0005	6.0
*Hypothalamus*		↑	−2	2	−13	212	<0.0001	9.7
*Midbrain*		↑/↓	2	−34	−1	132	<0.0001	6.6
*Orbital frontal cortex*	Left	↑	−38	30	−13	65	<0.0001	5.5
	Right	↑	40	38	−11	13	0.005	5.6
*Pregenual anterior cingulate cortex*	Left	↓	−12	52	3	103	<0.0001	5.0
	Right	↓	12	42	−5	55	0.0001	4.9
*Pallidum*	Left	↑	−16	−2	−1	4	0.02	2.9
	Right	↓	24	−12	−7	9	0.008	2.8
*Putamen*	Left	↓	−20	10	11	4	0.02	2.5
	Right	↓	14	12	−5	3	0.02	3.8

The F values of the peak voxel in the clusters are reported (F-peak) with corresponding MNI coordinates “x”, “y”, and “z”. All local maxima are significant at a height threshold of p_FWE_ < 0.05. FWE, family-wise error.

↑, and ↓ indicate elevated and reduced brain activity, respectively, after erythromycin compared to placebo infusion. ↑/↓ indicates activation and deactivation at the same peak but at different time points.

### Associations between appetite-related sensations, hedonic eating, and brain activity

#### Hunger and prospective food consumption

**Figure 5:**
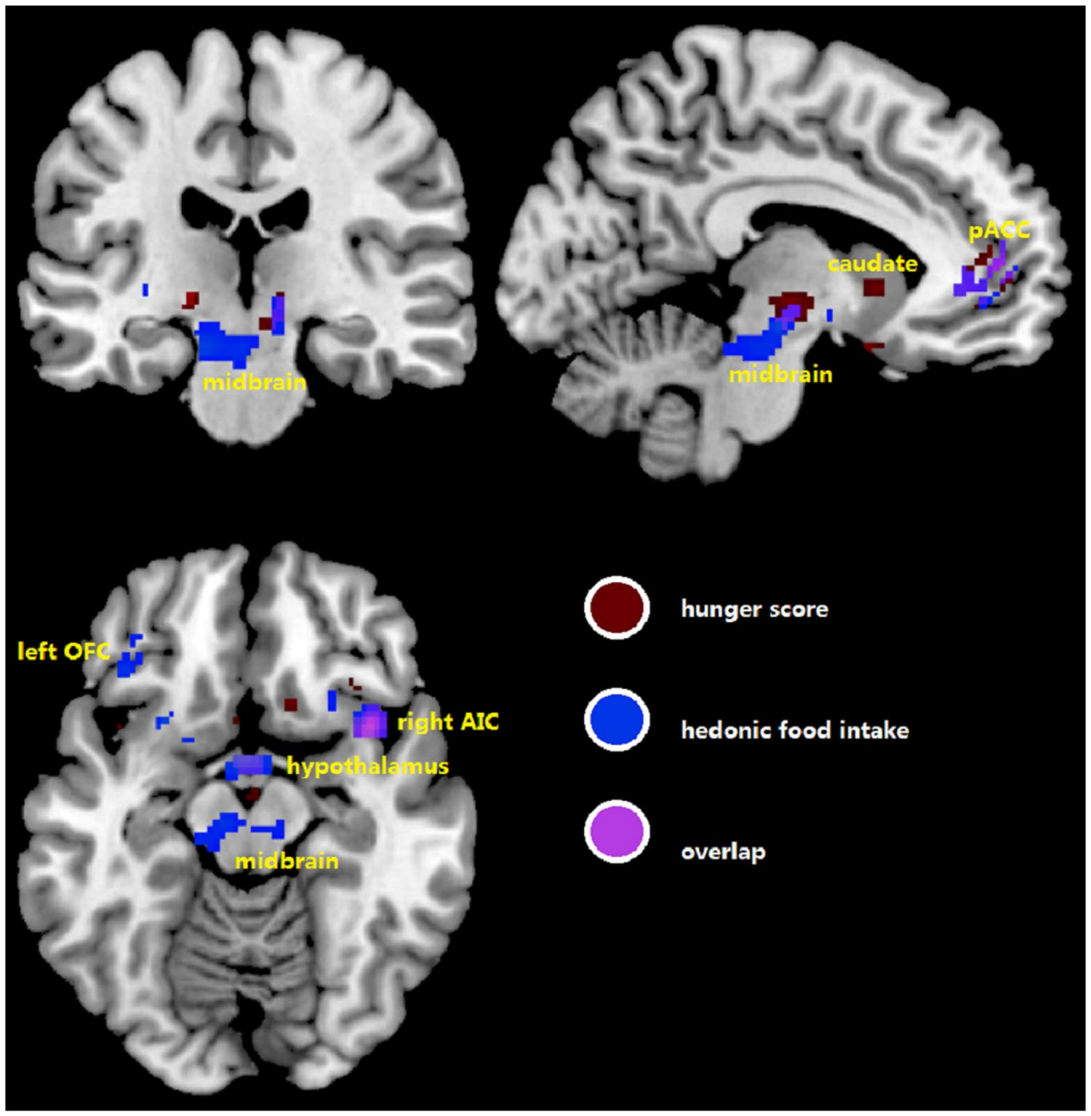
Overview of homeostatic and hedonic brain regions where differential activation by erythromycin compared to saline covaries with differences in hunger ratings (in brown) and hedonic food intake (in blue). pACC, perigenual anterior cingulate cortex; AIC, anterior insular cortex; OFC, orbitofrontal cortex.

The difference in hunger ratings between conditions covaried with the difference in BOLD response in AIC, caudate, OFC, pACC, and putamen bilaterally, left amygdala, left pallidum, hypothalamus, medulla, and midbrain, as shown in Fig. 5. Similar results were obtained with prospective food consumption ratings (details in Supplementary Table S1 and S2).

#### Hedonic food intake

Differences in hedonic food intake between the erythromycin and placebo conditions were predicted by differential brain responses in AIC, amygdala, OFC, pACC, and putamen bilaterally, right caudate, right pallidum, hypothalamus, medulla and midbrain, as shown in Fig. 5. (details in Supplementary Table S3).

### Associations between hormonal responses and brain activity

#### Octanoylated ghrelin

The difference in plasma octanoylated ghrelin levels between conditions covaried with the difference in BOLD response in AIC, amygdala, caudate, OFC, pACC, pallidum, and putamen bilaterally, hypothalamus, and medulla (details in Supplementary Table S4 and Supplementary Figure S1).

#### Blood glucose

The difference in blood glucose levels between conditions covaried with the difference in BOLD response in AIC, caudate, OFC, pACC, and putamen bilaterally, right nucleus accumbens, left amygdala, hypothalamus and midbrain (details in Supplementary Table S5 and Supplementary Figure S2).

#### Insulin

The difference in plasma insulin changes between conditions covaried with the difference in BOLD response in AIC, caudate, pACC, and putamen bilaterally, left amygdala and OFC, midbrain and medulla (details in Supplementary Table S6 and Supplementary Figure S3).

## Discussion

In the present study, we replicated the hunger-inducing effect of the motilin agonist erythromycin found in previous studies in healthy female volunteers by our group^7,8^. Further, we demonstrated for the first time that this leads to increased hedonic food intake 50 minutes after the start of the infusion (although it should be noted that only a non-significant trend was found, which may be due to the late administration of the test relative to infusion, see below). Finally and most importantly, we investigated the brain responses to the motilin agonist erythromycin using the pharmacological MRI design, which has been developed and widely used to measure the brain responses to drugs as well as nutrients in human^17–21^. We showed for the first time that the motilin receptor agonist, erythromycin, exerts its orexigenic effects by modulating the function of homeostatic and hedonic brain regions involved in the control of appetite and food intake, mediated by the brain-gut axis. Further, the differential response in these regions covaries with the increase in hunger ratings and predicts the subsequent increase in food intake after erythromycin versus placebo.

Regarding the stimulatory effect of a low dose of erythromycin on hunger and prospective food consumption ratings, we confirmed previous work by our group^7,8^. In line with these effects on subjective ratings, there was a trend towards more hedonic food intake after erythromycin infusion compared to placebo, with a small effect size (Cohen’s *d*: 0.44). This effect may not have reached full significance due to the rather long interval between the infusion and the task (because of the primary focus of this study on brain responses). Hence, this needs to be confirmed in studies testing hedonic food intake at 30–40 minutes after the start of the infusion (i.e. the moment where the difference in hunger ratings between both conditions was the largest in the present study). Nevertheless, these results are in agreement with our previous work showing that healthy volunteers requested a soup meal more often after i.v. erythromycin compared to saline infusion^7^. Interestingly, the responses in reward and homeostatic brain regions discussed below predicted the increase of hedonic food intake after erythromycin versus placebo infusion. Further, these brain regions mostly overlapped with the brain regions that covaried with the increase of hunger ratings. These findings indicate that the effect of erythromycin on reward and homeostatic brain regions mediated its hunger-inducing effect and therefore predicted the increase in hedonic food intake.

Among our predefined ROIs, the hypothalamus can be considered as the most important homeostatic region involved in the control of food intake^22^. We found enhanced activity after erythromycin administration in the hypothalamus, and this increase in activity covaried with the increase of subjective hunger and predicted the increase of *ad libitum* food intake after the erythromycin administration. Taken together, these findings suggest that the hypothalamus plays a key role in erythromycin-induced increases in hunger and food intake.

However, we also found that erythromycin activated important reward-related regions as the (ventral) midbrain (ventral tegmental area), ventral (nucleus accumbens) and dorsal striatum (caudate and putamen), amygdala, AIC, OFC, and ACC, all of which are key regions of the mesolimbic dopaminergic reward circuitry in the brain^1^. In fMRI studies, differences in responses to visual high caloric food stimuli (anticipatory reward) and/or receipt of rewarding food (consummatory reward) between normal weight and obese subjects were found in most of these reward-related regions, highlighting their important role in (dys)regulation of appetite and food intake in health and disorders of food intake^23^. In our study, we found associations between the differential brain responses in most of these mesolimbic reward system regions and the increase of hunger and prospective food consumption ratings after erythromycin versus saline administration, and the differential responses in these regions predicted differences in hedonic food intake. This indicates that not only an effect on homeostatic circuitry, but also on mesolimbic reward circuitry may be an important candidate brain-based mediator of the orexigenic effect of erythromycin.

It should be noted that the direction of the responses differed between these regions, with some of them showing an increase in activity in response to erythromycin, and others showing a decrease. Decrease of the BOLD signal probably indicates relatively smaller blood flow in these regions. In fact, a negative correlation between gamma-aminobutyric acid (GABA) concentration and BOLD signals has been demonstrated previously^24^. Therefore inhibitory neurotransmitters such as GABA might be involved in the response to erythromycin infusion. Little *et al*. found a 5 min increase followed by a decrease of the BOLD signal in medulla, pons, midbrain and hypothalamus in response to a bolus intragastric glucose infusion in a phMRI design^21^. They suggested that the decrease was driven by the increase of circulating glucose and insulin. In our study, the regions that showed a decreased BOLD signal in response to erythromycin infusion indeed covaried with the increase of glucose or insulin levels (see below), even though the regions differed from the ones found by Little *et al*. after intragastric glucose infusion (bilateral pACC, bilateral AIC, left amygdala and midbrain).

The exact gut-brain signaling mechanisms underlying the orexigenic effects of motilin and their neural basis had not been studied until now. However, Deloose *et al*.^7^ found that, at the dose which was also used in the present study, this effect is controlled via a cholinergic pathway at the level of the GI tract, as pre-treatment with atropine abolished the effect of erythromycin on both hunger and gastric phase III induction. In addition, old data indicate that erythromycin does only penetrate the blood-brain barrier to a very limited extent in a healthy population^25^. Therefore, we may speculate that gastric phase III contractions may generate an afferent signal conveyed from gut to brain by vagal and/or spinal afferents, which in turn influences brain activity. Due to incompatibility of the MR scanner environment with gastrointestinal manometry equipment, we were not able to measure gastric motility simultaneously during the scanning period. We were therefore not able to address direct correlations between erythromycin-induced gastric contractions and brain activity. However, although speculative, given the well-known role of the insula in processing (visceral) afferent interoceptive information, our finding of left ventral and right dorsal AIC activation after erythromycin infusion may be in line with this interpretation. More specifically, the AIC re-represents the interoceptive signals from the body and integrates them with emotional input, thereby playing a fundamental role in generating «interoceptive feelings» and, together with the ACC, motivational states, including pain and hunger^26^. The covariation we found between differential responses in these regions and subjective feelings (hunger, prospective food consumption) and eating behaviour (hedonic food intake) is in line with this interpretation. On the other hand, the motilin receptor is expressed in the human TE671 cell line, which originates from the cerebellum^27^. Moreover, it is probably also expressed on the vagus nerve as motilin increased gastric vagal afferent fiber activity *in vitro* in the *suncus murinus*^28^. This indicates that indirect (neural) or even direct (humoral) gut-brain signalling effects of motilin agonism cannot be excluded as a potential mechanism mediating the orexigenic effect of erythromycin on brain activity, in addition to motility-mediated effects described above. We observed differential timing of responses in different brain regions in this study, for example, the responses in the hypothalamus reached a peak at t = 20 min, whereas the responses in the bilateral OFC peaked at t = 30 min. It is therefore likely that brain responses in different regions were mediated by different or multiple pathways with a differential lag.

Given the observed erythromycin induced-effects on ghrelin, insulin, and glucose plasma levels and their covariation with the brain effects, these endocrine/metabolic effects may represent important alternative candidate neurohumoral gut-brain signals mediating the orexigenic effect of erythromycin. However, given the fact that hormone/glucose levels and brain responses are measured during the same timeframe, we cannot draw firm conclusion about the directionality of the associations, which renders the interpretation below inherently speculative.

Ghrelin is recognized as a key orexigenic hormone, and has a structural resemblance to motilin^29^. The fact that erythromycin administration not only prevented the increase in ghrelin levels over time seen after placebo infusion, but even induced a decrease in ghrelin levels (compared to the pre-infusion baseline) in this study argues against ghrelin as the key mediator of the orexigenic effect of motilin agonism. Therefore, the decrease in ghrelin could rather be the consequence of a negative feedback mechanism resulting from the erythromycin infusion (through peripheral or top-down brain-gut signaling mechanisms triggered by the brain response to motilin agonism). An inhibition of the adrenergic pathway controlling ghrelin release might be a possibility^30^.

We also found an increase of blood glucose and plasma insulin levels in response to erythromycin infusion. Suzuki *et al*. showed^16^ that in dogs exogenous motilin induced the release of insulin through vagal cholinergic pathways in the fasted state, without triggering any change in blood glucose levels. They also found that, in the interdigestive state, plasma insulin concentrations peaked during early gastric phase III. This is in line with our results showing an early peak (t = 20–30 min) in plasma insulin levels after erythromycin infusion. Indeed, Ueno *et al*. have already shown that administration of erythromycin increases the release of insulin in human subjects in fasting state^15^. However, in our study we also found an increase of blood glucose together with an increase of plasma insulin after erythromycin infusion 2 hours after a standard meal. This can potentially be explained by the difference between fasted state and interprandial state, as erythromycin is well-known for being a prokinetic agent and hence improves gastric emptying^31^. In our study, erythromycin was infused 2 hours after a small meal (275 kcal). In healthy humans, gastric half emptying time of a similar solid meal (250 kcal) varies between 20 and 118 min^8^. It is therefore possible that the increase in blood glucose levels after erythromycin infusion is caused by emptying of food remnants from the stomach into the small intestine. In this case, the observed increase in insulin levels after erythromycin infusion could be secondary to the increase in glucose levels.

In addition, the change of blood glucose and plasma insulin levels covaried with brain responses in homeostatic and hedonic regions. Interestingly, there were some remarkable differences between the regions that covaried with glucose and those with insulin. Changes of blood glucose covaried most strongly with the homeostatic hypothalamus, and a few smaller clusters in reward-related regions (bilateral AIC, pACC, caudate and left amygdala, supplementary Table S5). Changes of plasma insulin, on the contrary, covaried most strongly with reward related regions (bilateral AIC, pACC, caudate, putamen and left amygdala, supplementary Table S6), but not with the hypothalamus. Responses to glucose administration have been shown in the hypothalamus^21,32^, midbrain, insula and putamen^21^ in MRI studies. These regions overlap with the regions that covaried with the change of blood glucose levels in response to erythromycin administration in our study. Not much is known about brain responses to exogenous insulin, as this requires a hyperinsulinemic-euglycemic clamp technique^33^. One possible explanation is that in our study, the increase of insulin was induced by erythromycin infusion, whereas erythromycin *per se* activated the hypothalamus. Therefore the covariation might be neutralized by the activation effect of erythromycin and the deactivation induced by glucose. More generally, it remains unclear whether the effects on glucose and/or insulin represent the true gut-brain signaling mediators of the observed orexigenic effect of erythromycin infusion, or are rather epiphenomena. As for ghrelin, the direction of the effects of erythromycin on glucose (increase) and hunger (increase) may argue againt a true mediating role, as, if anything, an increase in blood glucose would be expected to reduce hunger.

Some limitations of our study require consideration. First, gastric motility and gastric emptying were not measured in this study, because of the uncompatibility of the high-resolution manometry probe with the magnetic field of the megnatic resonance imaing (MRI) scanner environment, as mentioned earlier. Second, due to the limitation of the spatial resolution of functional MRI (fMRI), we were not able to differentiate various nuclei in the brainstem. Third, the sample size was relatively small, due to the complexity of the study design and high costs of the fMRI scans. To achieve a homogeneous sample and avoid potentially confounding sex differences, we only included healthy women, which implies the limitation that the present results cannot be extrapolated to men.

In summary, the motilin agonist, erythromycin, increased hunger and hedonic food intake and attenuated the secretion of ghrelin in healthy female volunteers. Homeostatic brain regions, more specifically the hypothalamus, were activated in response to erythromycin infusion, and the activation of hypothalamus covaried with the increase of hunger and predicted the increase of hedonic food intake. Further, brain responses to erythromycin were also found in several key reward regions. The responses in most of these regions (including bilateral AIC, left amygdala, bilateral caudate, bilateral OFC, and bilateral ACC) covaried with the increase of hunger, and, in most of the cases, also predicted the increase of hedonic food intake after erythromycin infusion. These results confirm recent behavioural findings identifying motilin as a novel key orexigenic hormone in humans, and identify the gut-brain signaling mechanisms underlying its effect. Therefore, although further research is needed, we believe these results may inspire future drug development to improve dysregulation of appetite and food intake in patients populations characterized by loss of appetite and weight loss, including functional dyspepsia, anorexia nervosa and/or cachexia due to organic conditions.

## Materials and Methods

### Eligibility criteria for participants

Right-handed, normal weight, healthy women (18–65 years) were included, to avoid sex as a potential confounder. Exclusion criteria included smoking, substance abuse, regular intake of medications with an exception of oral contraception pills, chronic medical illness or illnesses affecting the gastrointestinal, cardiovascular, or nervous systems, chronic pain or psychiatric disorders (including major depression and chronic health conditions except controlled hypertension), pregnancy and lactation, and any contraindication to MRI) (e.g. claustrophobia, non-removable metal devices). None of the subjects had a history of lactose or gluten intolerance or dietary restrictions. Sample size was chosen based on previous work on the effects of erythromycin on hunger ratings^4^, given the lack of evidence to estimate the effect size at the brain level, not permitting any formal power calculation.

### Study design and experimental procedure

This randomized (counterbalanced), placebo-controlled, single-blind, cross-over study was approved by the Medical Ethics Committee of the University Hospitals Leuven, Belgium (ML10263, 15-May-2014), registered at ClinicalTrials.gov (NCT02212821, 21 Mar 2014) and performed in the University Hospital Gasthuisberg, Leuven, Belgium, in accordance with the Declaration of Helsinki, including written informed consent.

Subjects were studied on two separate occasions, at least one week apart. At 08.30 AM after a 12-hour overnight fast, participants received a 275 kCal standardized breakfast [2 slices of toasted white bread (45 g), 2 slices of cheese (80 g), 1 tablespoon of jam (25 g) and 200 mL orange juice (200 g)]^34^ to avoid a potential ceiling effect on hunger ratings if subjects would have been studied fasted. After breakfast, two intravenous catheters were placed for blood collection and single-blinded erythromycin/saline (placebo) infusion, respectively. One hundred and fifteen minutes after breakfast, participants entered the MR scanner. After a 10 min adaptation period, the MRI scan started and lasted for 50 min in total. After 10 min baseline scanning, either saline or erythromycin [40 mg erythromycin lactobionate (Amdipharm Limited, Dublin, Ireland) dissolved in 100 mL 0.9% NaCl] were infused for 20 min (5 mL/min). This dose of erythromycin has been applied in previous studies of our research group, and it was shown to reliably induce gastric phase III and proved to be safe and well tolerated^7,8^. The timing of erythromycin infusion (135 min after breakfast consumption) was chosen since erythromycin administration 2 hours after a 250 kcal meal increases hunger scores^8^. Blood samples for hormone analysis and visual analogue scales (VAS) for appetite-related sensations were collected at regular time intervals before (baseline) and after start of the infusion. Immediately after the end of scanning, subjects left the scanner for a 10 min break. Finally, hedonic food intake was measured using an *ad libitum* chocolate milkshake drinking test.

### Blood sample processing and laboratory analysis

Blood samples were immediately processed on ice to measure plasma octanoylated ghrelin, motilin, and insulin using radioimmunoassay (ghrelin/motilin), or enzyme-lined immunosorbant assay (insulin) (details in *Supplement)*.

### Appetite-related sensations assessment

Validated computer-based VAS were used to rate the subjective sensations of hunger, prospective food consumption, fullness, satiety and nausea (details in *Supplement)*.

### Milkshake drinking task

Subjects were instructed to drink chocolate milkshake at their own pace until satiation. The amount of milkshake drunk was determined as a measurement of hedonic eating behavior. The milkshake used in the drinking task was adapted from Stice *et al*. with Belgian brands^35^. Subjects were instructed to drink the chocolate milkshake (4 scoops of IJsboerke vanilla ice cream, 355 mL of 2% milk, and 2 tablespoons of Imperial chocolate syrup, 270 kcal, 13.5 g fat, and 28 g sugar per 150 mL) from a 200 mL glass, at their own pace until they felt comfortably satiated, to measure hedonic eating behavior. The glass was immediately refilled when it was empty. The amount of milkshake before and after the task was weighted on a scale to calculate the amount of milkshake drunk by the volunteer. At the end of the task, participants were asked “How much do you like the milkshake”, with anchors “not at all” and “extremely”. All subjects liked the milkshake (VAS liking of milkshake: 74 ± 3). Therefore, the milkshake drink was considered rewarding to all the subjects.

### MRI data acquisition and preprocessing

Briefly, standard BOLD signal fMRI data were collected with a gradient echo planar imaging (EPI) sequence on a 3.0 Tesla MRI scanner (Philips Medical Systems, Best, Netherlands) with a 32 channel head coil. In addition, a high-resolution structural image was obtained for coregistration purposes. Standard preprocessing steps (realignement, coregistration, spatial normalization, and smoothing) were applied to the data.

For the functional scan, a total of 1200 volumes of 46 slices (2.7 mm thickness, interslice gap of 0.3 mm) were acquired continuously per visit for a total examination duration of 50 min (TE/TR = 30/2500 ms, flip angle (FA) = 90°, matrix size = 88 × 85, field of view = 21 × 21 cm). After the functional scan, a high-resolution structural scan was acquired (46 2.7-mm-thick near-axial slices with 0.3 mm gap, TR = 9.6 ms, TE = 4.6 ms, FA = 90 °, matrix size = 256 × 256, field of view = 25 × 25 cm).

Preprocessing was performed as follows. Two-pass realignment of the time series image volumes was followed by co-registration to the high-resolution structural image acquired in each session. The high-resolution image was used for each participant as reference for the spatial normalization to the EPI template image supplied with SPM. Spatial smoothing (8 mm FWHM Gaussian kernel) was afterwards applied to the normalized images to improve the signal to noise ratio in the data.

### Statistical analysis

#### Appetite-related sensations, hormone responses, and hedonic eating

Analyses were done using SAS 9.3 (SAS Institute, Cary, NC, USA). Data were reported as mean ± SEM. Significance was set at p ≤ 0.05, and 0.05 < p < 0.10 was considered a trend. Linear mixed models were performed on delta values (change from pre-infusion baseline), with main effects of time and condition (both within-subject effects) as well as the time-by-condition interaction effect. Interaction effects were followed up by *post-hoc* paired t-tests comparing both conditions at time points t = 30–40 min separately based on our hypothesis [two-tailed, with stepdown Bonferroni (Holm) correction for multiple testing]. Hedonic eating (amount of milkshake drunk) was compared between both conditions using a paired Student’s t-test.

Based on the timing of phase III onset after erythromycin infusion in our previous studies in the fasted and interprandial state^7,8^, we hypothesized that appetite-related sensations would differ between erythromycin and placebo from 30 min after infusion. This hypothesis was tested using planned contrast analysis on the estimates from the mixed models comparing the difference of the average change in appetite-related sensations from baseline over the 30, 35, and 40 min time bins between conditions. Further, as we had a specific *a priori* hypothesis about the direction of the effect for appetite-related sensations and hedonic eating based on previous work^7,8^ [*higher* hunger and prospective food consumption ratings (and lower fullness and satiety ratings) as well as *increased* hedonic eating after erythromycin compared to placebo], we performed one-tailed testing on these variables.

#### MRI data analysis

Data were analyzed with SPM12 (Wellcome Department of Cognitive Neurology, London, UK) using a previously developed phMRI analysis method^17–21,36^.

At *first (individual) level*, the 50 pre-infusion volumes were used as baseline for each condition. In order to compare the BOLD signal changes relative to baseline between conditions, all post-infusion volumes (from t = 0 to 40) were divided into 1-minute time bins (24 volumes per time bin). A high pass filter of 2400 s (because the infusion lasted 20 min) was used to minimize the influence of very low frequency noise in the BOLD signal. The mean time series of white matter and cerebrospinal fluid were entered in the design matrix as regressors of no interest together with realignment parameters, to correct for putative differences in scanner drift and subject movement between conditions, respectively. One t-contrast per time bin was calculated to compare the brain response (relative to pre-infusion baseline) between erythromycin and placebo.

*Second (group) level* voxelwise analysis was performed on the t-contrasts from the first level analysis. For this purpose, a within-subject one-way ANOVA model was applied to compare the difference in signal change (relative to baseline) between conditions over time bins at the group level, with the condition-by-time interaction effect being the effect of interest. The resulting statistical parametric map was thresholded at p < 0.05 family-wise error corrected at the voxel level in a pre-defined mask.

Second level analysis was performed in the mask of *a priori* regions of interest (ROIs), based on previous knowledge of brain regions involved in hedonic^37^ and homeostatic^17^ control of appetite and food intake. These ROIs were anatomatically defined based on atlases provided in the SPM12 toolbox WFU Pickatlas^38^, including anterior insula cortex (AIC), lateral and medial parts of orbital frontal cortex (OFC), amygdala, hypothalamus, medulla – nucleus tractus solitarii (NTS), midbrain, pregenual anterior cingulate cortex (pACC), pallidum, dorsal striatum (caudate head and body, and putamen), and ventral striatum (nucleus accumbens). For each region and each time bin, the contrast estimates reflecting the difference in brain response (relative to baseline) between both conditions were extracted with the SPM12 tool box MarsBar for visualization purposes.

#### Associations between appetite-related sensations, hormonal responses, hedonic eating, and brain activity

To test in which regions (within the abovementioned ROI mask) the difference in appetite-related sensations, hormonal responses, and hedonic eating between the conditions covaried with the difference in brain responses, the differences in appetite-related sensations, hormone levels (expressed as absolute change from baseline), and hedonic food intake were used to weigh the first level contrasts in the corresponding time bins. For this purpose, appetite-related sensation ratings and hormone levels were linearly interpolated to match the number of time bins in the first level phMRI analysis in SPM. For hedonic eating, the single delta value reflecting the difference in milkshake intake between both conditions was used to weigh the first level contrast in each timebin. The same statistical threshold as for the analysis comparing both conditions was used.

### Data availability statement

The datasets generated during and anlysed during the current study are available from the corresponding author on reasonable request.

## Electronic supplementary material

Supplementary Information
