# Supplementary material for: The motilin agonist erythromycin increases hunger by modulating homeostatic and hedonic brain circuits in healthy women: a randomized, placebo-controlled study
Source: Sci Rep. 2018 Jan 29;8:1819. doi: 10.1038/s41598-018-19444-5 (PMC5789052; doi:10.1038/s41598-018-19444-5)
Supplement: Supplementary file 1 — Supplementary Information [file 41598_2018_19444_MOESM1_ESM.doc]

**Title**

## The motilin agonist erythromycin increases hunger by interfering with homeostatic and hedonic brain circuits in healthy women: a randomized, placebo-controlled study

**Author list and affiliations**

Dongxing Zhao^1,+^, Anne Christin Meyer-Gerspach^1,2,+^, Eveline Deloose^1^, Julie Iven^1^, Nathalie Weltens^1^, Inge Depoortere^1^, Owen O’daly^3^, Jan Tack^1^, Lukas Van Oudenhove^1,*^

^+^equal contribution

^1^Translational Research Center for Gastrointestinal Disorders, Catholic University of Leuven, Leuven, Belgium

^2^Department of Research, Clara Hospital Basel, Basel, Switzerland

^3^King’s college London’s Institute of Psychiatry, London, UK

## *Corresponding Author Name

Prof. Dr. Lukas Van Oudenhove

Laboratory for Brain-Gut Axis Studies (LaBGAS)

Translational Research Center for Gastrointestinal Disorders (TARGID)

Catholic University of Leuven

B-3000 Leuven, Belgium

Phone: +32 16 33 01 47

Fax: +32 16 34 59 39

E-mail: lukas.vanoudenhove@kuleuven.be

## SUPPLEMENTARY INFORMATION

### Supplementary Materials and Methods

### Blood sample processing and laboratory analysis

Ghrelin blood samples were collected on ice in EDTA tubes (Becton, Dickinson and Company, Franklin Lakes, NJ, USA) supplemented with 500 kIU/mL aprotinin (Roche Applied Science, Penzberg, Germany) and phenylmethansulfonylfluoride (PMSF; 0.57mM; Sigma-Aldrich, Steinheim, Germany). Motilin blood samples were collected on ice in lithium heparine tubes (Becton, Dickinson and Company, Franklin Lakes, NJ, USA) containing 500 kIU/mL aprotinin. Insulin blood samples were collected on ice in EDTA tubes supplemented with 500 kIU/mL aprotinin and a dipeptidylpeptidase-4 (DPP-4) inhibitor (10 μL/mL; Millipore Corporation, St. Charles, Missouri, USA). Tubes were centrifuged at 4° C at 3000 rpm for 10 min and plasma samples were aliquoted. Plasma samples for ghrelin measurements were immediately acidified (10%) with 1N HCL and extracted on a Sep-Pak C18 cartridge (Waters Corporation, Milford, Massachusetts, USA) and dried in a speedvac^1^. All samples were stored at -80°C until analysis of plasma octanoylated ghrelin, motilin, and insulin. Blood glucose was measured immediately in whole blood with a glucometer (Menarini Diagnostics, Zaventem, Belgium).

*Plasma octanoylated ghrelin* was measured by radioimmunoassay with 125I [Tyr24] human ghrelin [1-23] as tracer and a rabbit antibody against human ghrelin [1-8] (final dilution 1/100000), which does not cross-react with desoctanoylated ghrelin, as described previously in more detail.^1^

*Plasma motilin* was measured as previously described using 125I [Nle13] human motilin as tracer and rabbit anti-human Nle13 motilin antibody (final dilution 1/18000)^2^.

*Plasma insulin* was measured with a commercially available ELISA kit (EZHIASF-14K; EMD Millipore Corporation, Billerica, MA, USA). The assay ranged from 2 µU/mL to 200 µU/mL human insulin. The lowest insulin concentration detected by this assay is 0.85 μU/mL in a 20 μL plasma sample. The intraassay coefficient of variation is 1.8% and the interassay coefficient of variation 6.6%.

### Appetite-related sensations assessment

Computer-based visual analog scales (VAS) were used to rate the subjective sensations of hunger, prospective food consumption, fullness, satiety and nausea. The scales have been previously validated^3^, ^4^. Subjects were instructed to indicate their subjective sensations at the present time point by moving a mark on the screen using an MRI compatible button box (in 5 mm steps). In order to correctly allocate the VAS score to the corresponding volumes during scanning in both conditions, the duration of each VAS was fixed to 7 seconds. In addition, the mark was always reset to the middle of the vertical line at the beginning of each VAS question.

### Supplementary Results

### Appetite-related sensations

#### Hunger and prospective food consumption

Scores of both hunger and prospective food consumption increased regardless of erythromycin or placebo infusion (main effect of time, F_7,84_=3.66 p=0.002, F_7,178_=3.88 p<0.001, respectively). No effect of erythromycin infusion was found on increase in hunger or prospective food consumption ratings over the entire time period from the beginning of infusion (main effect of condition, F_1,12_=2.26 p=0.16, F_1,12_=2.26 p=0.16, respectively). No interaction effect between time and condition was observed (time-by-condition interaction, F_7,177_=0.87 p=0.53, F_7,177_=0.50 p=0.83, respectively).

*Satiety and Fullness*

Scores of both satiety and fullness decreased regardless erythromycin or placebo infusion (main effect of time, F_7,177_=3.27 p=0.002, F_7,177_=6.51 p<0.0001, respectively). No effect of erythromycin infusion was found on change in satiety or fullness ratings over the entire time period from the beginning of infusion (main effect of condition F_1,177_=0.14 p=0.71, F_1,177_=0.02 p=0.88, respectively). No interaction effect between time and condition was observed (time-by-condition interaction, F_7,176_=1.06 p=0.39, F_7,176_=0.75 p=0.63, respectively).

## Supplementary Tables

| *Regions* | Side | x  (mm) | y  (mm) | z  (mm) | Cluster volume | p(FWE-corrected) | F-peak |
| --- | --- | --- | --- | --- | --- | --- | --- |
| *AIC* | Left | -26 | 20 | -3 | 8 | 0.008 | 3.4 |
|  | Right | 38 | 12 | -13 | 90 | <0.0001 | 6.9 |
| *Amygdala* | Left | -30 | -4 | -21 | 5 | 0.01 | 3.6 |
| *Caudate* | Left | -8 | 12 | 1 | 87 | <0.0001 | 3.8 |
|  | Right | 16 | 12 | 15 | 238 | <0.0001 | 7.2 |
| *Hypothalamus* |  | -2 | 0 | -7 | 61 | <0.0001 | 4.0 |
| *Medulla* |  | -6 | -28 | -43 | 2 | 0.02 | 3.0 |
| *Midbrain* |  | 6 | -30 | -1 | 117 | <0.0001 | 9.1 |
| *OFC* | Left | -36 | 32 | -17 | 16 | 0.003 | 5.1 |
|  | Right | 18 | 12 | -21 | 22 | 0.002 | 4.2 |
| *pACC* | Left | -8 | 50 | 7 | 137 | <0.0001 | 5.6 |
|  | Right | 12 | 40 | 27 | 10 | 0.006 | 3.7 |
| *Pallidum* | Left | -26 | -18 | -5 | 8 | 0.008 | 3.6 |
| *Putamen* | Left | -24 | 0 | -1 | 52 | 0.0001 | 3.1 |
|  | Right | 20 | 10 | 11 | 1 | 0.03 | 2.6 |

***Supplementary Table S1. Regions when differences in brain responses to erythromycin versus saline covaried with differences in hunger ratings.*** *The F values of the peak voxel in the clusters are reported (F-peak) with corresponding MNI coordinates “x”, “y”, and “z”. All local maxima are significant at a height threshold of p_FWE_ < 0.05. FWE, family-wise error. pACC, perigenual anterior cingulate cortex; AIC, anterior insular cortex; OFC, orbitofrontal cortex.*

| *Regions* | Side | x  (mm) | y  (mm) | z  (mm) | Cluster volume | p(FWE-corrected) | F-peak |
| --- | --- | --- | --- | --- | --- | --- | --- |
| *AIC* | Left | -34 | 18 | 3 | 4 | 0.02 | 2.5 |
|  | Right | 38 | 14 | -13 | 46 | 0.0002 | 4.7 |
| *Amygdala* | Left | -30 | -4 | -21 | 5 | 0.02 | 3.0 |
|  | Right | 22 | -10 | -11 | 5 | 0.02 | 3.1 |
| *Caudate* | Left | -12 | 10 | 3 | 250 | <0.0001 | 4.9 |
|  | Right | 14 | 12 | 15 | 398 | <0.0001 | 6.9 |
| *Midbrain* |  | 4 | -32 | 1 | 108 | <0.0001 | 7.1 |
| *OFC* | Left | -34 | 34 | -15 | 41 | 0.0004 | 6.2 |
|  | Right | 20 | 26 | -15 | 3 | 0.02 | 2.7 |
| *pACC* | Left | -6 | 42 | -1 | 66 | <0.0001 | 3.8 |
|  | Right | 12 | 44 | 21 | 29 | 0.001 | 4.5 |
| *Pallidum* | Left | -24 | -18 | -1 | 1 | 0.03 | 2.4 |
| *Putamen* | Left | -28 | -12 | 15 | 4 | 0.02 | 2.9 |

***Supplementary Table S2. Regions when differences in brain responses to erythromycin versus saline covaried with differences in prospective food comsumption ratings.*** *The F values of the peak voxel in the clusters are reported (F-peak) with corresponding MNI coordinates “x”, “y”, and “z”. All local maxima are significant at a height threshold of p_FWE_ < 0.05. FWE, family-wise error. pACC, perigenual anterior cingulate cortex; AIC, anterior insular cortex; OFC, orbitofrontal cortex.*

| *Regions* | Side | x  (mm) | y  (mm) | z  (mm) | Cluster volume | p(FWE-corrected) | F-peak |
| --- | --- | --- | --- | --- | --- | --- | --- |
| *Amygdala* | Left | -30 | -4 | -23 | 22 | <0.0001 | 5.7 |
|  | Right | 28 | -6 | -13 | 4 | <0.0001 | 3.3 |
| *AIC* | Left | -26 | 12 | -15 | 33 | <0.0001 | 10.2 |
|  | Right | 38 | 12 | -13 | 90 | <0.0001 | 6.9 |
| *Caudate* | Right | 14 | 12 | 17 | 35 | <0.0001 | 6.0 |
| *Hypothalamus* |  | -2 | 2 | -13 | 106 | <0.0001 | 7.8 |
| *Medulla* |  | -6 | -28 | -43 | 2 | 0.0002 | 3.0 |
| *Midbrain* |  | -14 | -24 | -17 | 279 | <0.0001 | 7.5 |
| *Midbrain* |  | 4 | -36 | -5 | 157 | <0.0001 | 8.7 |
| *OFC* | Left | -38 | 30 | -13 | 39 | <0.0001 | 4.7 |
|  | Right | 40 | 38 | -11 | 7 | <0.0001 | 4.3 |
| *pACC* | Left | -10 | 48 | -1 | 144 | <0.0001 | 4.0 |
|  | Right | 12 | 44 | -5 | 97 | <0.0001 | 4.4 |
| *Pallidum* | Right | 24 | -14 | -7 | 2 | 0.002 | 2.7 |
| *Putamen* | Left | -20 | 12 | -11 | 30 | <0.0001 | 4.7 |
|  | Right | 14 | 12 | -5 | 9 | <0.0001 | 4.1 |

***Supplementary Table S3. Regions when differences in brain responses to erythromycin versus saline covaried with differences in hedonic food intake.*** *The F values of the peak voxel in the clusters are reported (F-peak) with corresponding MNI coordinates “x”, “y”, and “z”. All local maxima are significant at a height threshold of p_FWE_ < 0.05. FWE, family-wise error. pACC, perigenual anterior cingulate cortex; AIC, anterior insular cortex; OFC, orbitofrontal cortex.*

| *Regions* | Side | x  (mm) | y  (mm) | z  (mm) | Cluster volume | p(FWE-corrected) | F-peak |
| --- | --- | --- | --- | --- | --- | --- | --- |
| *AIC* | Left | -26 | 22 | -13 | 9 | <0.0001 | 4.5 |
|  | Right | 28 | 28 | 3 | 7 | <0.0001 | 4.3 |
| *Amygdala* | Left | -30 | -4 | -23 | 3 | <0.0001 | 3.0 |
|  | Right | 24 | 2 | -19 | 2 | 0.02 | 2.5 |
| *Caudate* | Left | -6 | 4 | -3 | 44 | <0.0001 | 6.1 |
|  | Right | 14 | 12 | 17 | 12 | <0.0001 | 3.1 |
| *Hypothalamus* |  | -2 | 0 | -5 | 352 | <0.0001 | 6.2 |
| *Medulla* |  | 0 | -34 | -47 | 101 | <0.0001 | 3.7 |
| *Midbrain* |  | 6 | -30 | -3 | 86 | <0.0001 | 3.3 |
| *OFC* | Right | 22 | 36 | -11 | 36 | <0.0001 | 4.1 |
|  | Left | -16 | 12 | -17 | 37 | <0.0001 | 4.7 |
| *pACC* | Left | -4 | 50 | -3 | 54 | <0.0001 | 3.6 |
|  | Right | 12 | 40 | -5 | 59 | <0.0001 | 4.8 |
| *Pallidum* | Left | -16 | -2 | -1 | 29 | <0.0001 | 4.2 |
|  | Right | 16 | 0 | -7 | 12 | <0.0001 | 5.0 |
| *Putamen* | Left | -28 | -10 | 15 | 61 | <0.0001 | 3.1 |
|  | Right | 16 | 0 | 7 | 4 | 0.002 | 2.7 |

***Supplementary Table S4. Regions when differences in brain responses to erythromycin versus saline covaried with differences in plasma octanoylated ghrelin.*** *The F values of the peak voxel in the clusters are reported (F-peak) with corresponding MNI coordinates “x”, “y”, and “z”. All local maxima are significant at a height threshold of p_FWE_ < 0.05. FWE, family-wise error. pACC, perigenual anterior cingulate cortex; AIC, anterior insular cortex; OFC, orbitofrontal cortex.*

| *Region* | Side | x  (mm) | y  (mm) | z  (mm) | Cluster volume | p(FWE-corrected) | F-peak |
| --- | --- | --- | --- | --- | --- | --- | --- |
| *AIC* | Left | -26 | 20 | -3 | 2 | 0.001 | 2.8 |
|  | Right | 26 | 22 | -7 | 1 | 0.02 | 2.5 |
| *Nucleus Accumbens* | Right | 14 | 14 | -11 | 22 | <0.0001 | 3.2 |
| *Amygdala* | Left | -18 | -2 | -27 | 2 | <0.0001 | 3.1 |
| *Caudate* | Left | -12 | -14 | 21 | 17 | <0.0001 | 3.1 |
|  | Right | 12 | -6 | 23 | 1 | 0.047 | 2.4 |
| *Hypothalamus* |  | -2 | -2 | -19 | 452 | <0.0001 | 5.3 |
| *Midbrain* |  | -14 | -14 | -3 | 15 | <0.0001 | 4.2 |
| *OFC* | Left | -24 | 12 | -25 | 36 | <0.0001 | 5.4 |
|  | Right | 36 | 38 | -9 | 15 | <0.0001 | 6.5 |
| *pACC* | Left | -12 | 40 | -9 | 14 | <0.0001 | 3.1 |
|  | Right | 2 | 20 | -11 | 19 | <0.0001 | 3.8 |
| *Putamen* | Left | -22 | 16 | -5 | 24 | <0.0001 | 3.5 |
|  | right | 26 | 0 | 15 | 17 | <0.0001 | 3.9 |

***Supplementary Table S5. Regions where differences in brain responses to erythromycin versus saline covaried with differences in blood glucose.*** *The F values of the peak voxel in the clusters are reported (F-peak) with corresponding MNI coordinates “x”, “y”, and “z”. All local maxima are significant at a height threshold of p_FWE_ < 0.05. FWE, family-wise error. pACC, perigenual anterior cingulate cortex; AIC, anterior insular cortex; OFC, orbitofrontal cortex.*

| *Region* | Side | x  (mm) | y  (mm) | z  (mm) | Cluster volume | p(FWE-corrected) | F-peak |
| --- | --- | --- | --- | --- | --- | --- | --- |
| *AIC* | Left | -38 | 14 | -13 | 303 | <0.0001 | 9.4 |
|  | Right | 40 | 16 | -9 | 302 | <0.0001 | 6.0 |
| *Amygdala* | Left | -22 | -6 | -21 | 8 | 0.009 | 2.9 |
| *Caudate* | Left | -6 | 4 | 15 | 72 | <0.0001 | 6.5 |
|  | Right | 6 | 6 | 15 | 71 | <0.0001 | 6.6 |
| *Midbrain* |  | 2 | -20 | -23 | 428 | <0.0001 | 5.6 |
| *Medulla* |  | 6 | -32 | -45 | 81 | <0.0001 | 7.5 |
| *Putamen* | Left | -24 | 6 | -7 | 60 | <0.0001 | 5.0 |
|  | Right | 28 | -14 | 11 | 270 | <0.0001 | 5.2 |
| *pACC* | Left | -2 | 38 | 15 | 203 | <0.0001 | 5.6 |
|  | Right | 4 | 38 | 17 | 210 | <0.0001 | 7.1 |
| *OFC* | Left | -32 | 34 | -13 | 34 | 0.0006 | 7.0 |

***Supplementary Table S6. Regions when differences in brain responses to erythromycin versus placebo covaried with differences in plasma insulin.*** *The F values of the peak voxel in the clusters are reported (F-peak) with corresponding MNI coordinates “x”, “y”, and “z”. All local maxima are significant at a height threshold of p_FWE_ < 0.05. FWE, family-wise error. pACC, perigenual anterior cingulate cortex; AIC, anterior insular cortex; OFC, orbitofrontal cortex.*

## Supplementary Figures


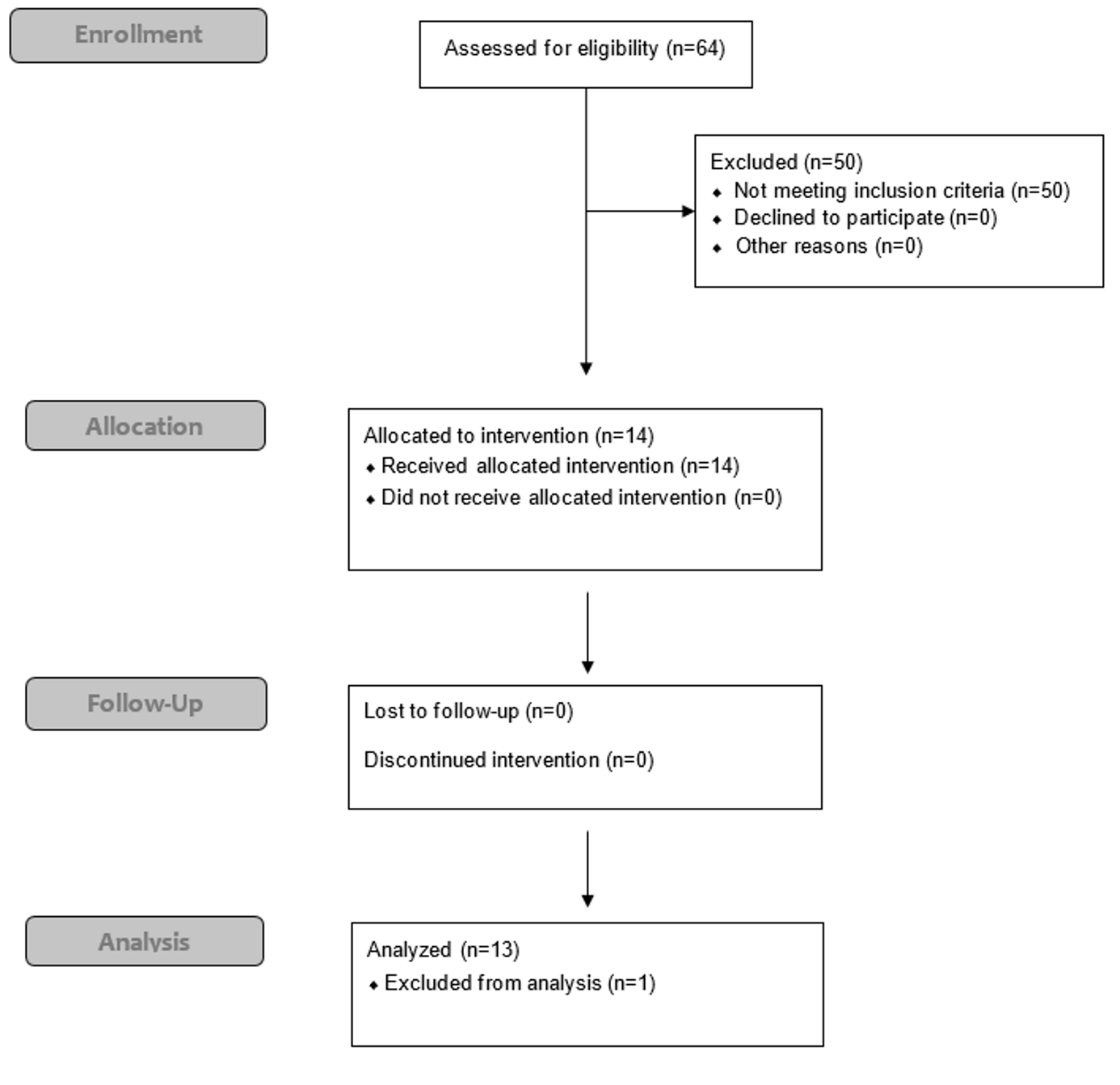


***Supplementary Figure S1. Overview of the recruitment procedure and flow of participants.***

**
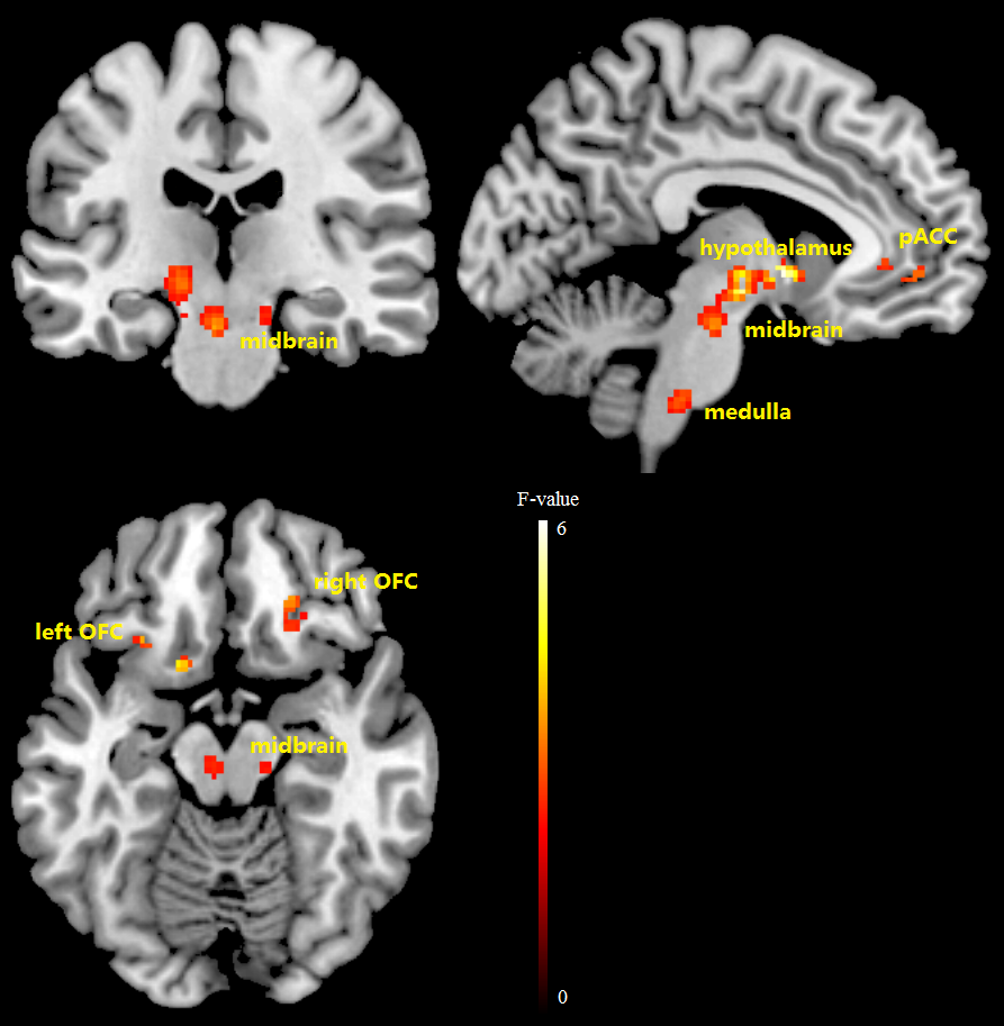
**

***Supplementary Figure S2. Brain regions where differential activation by erythromycin compared to saline covaries with differences in plasma octanoylated ghrelin levels.*** *Color bar represents F-values. pACC, perigenual anterior cingulate cortex; OFC, orbitofrontal cortex.*

***
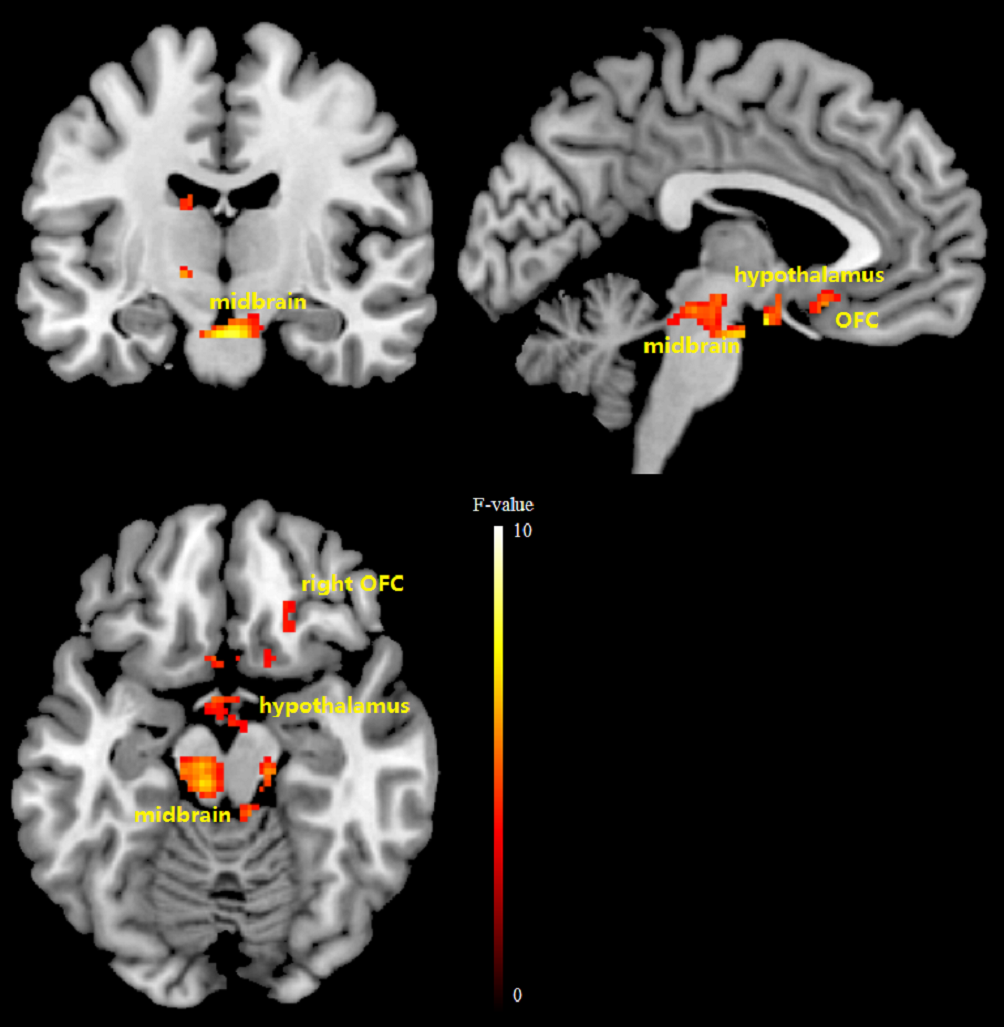
***

***Supplementary Figure S3. Brain regions where differential activation by erythromycin compared to saline covaries with differences in blood glucose levels.*** *Color bar represents F-values. OFC, orbitofrontal cortex.*

***
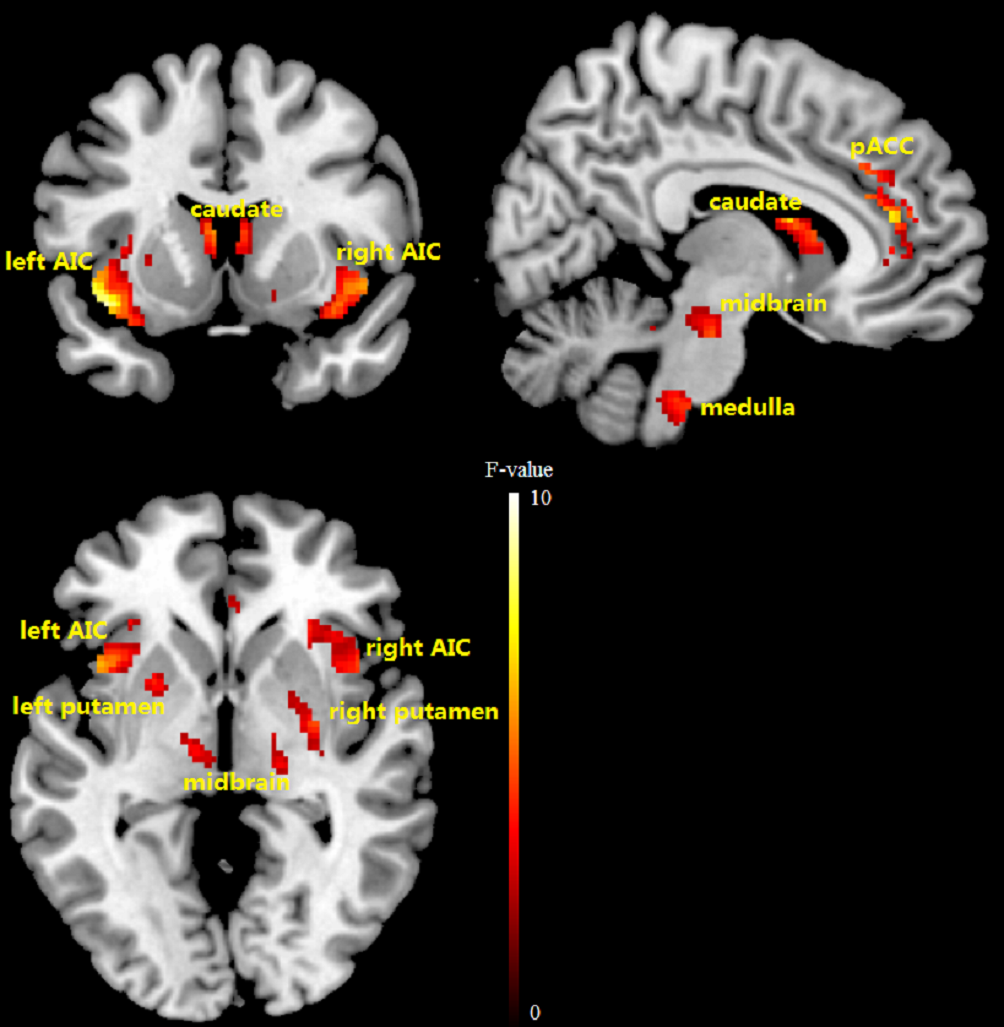
***

***Supplementary Figure S4. Brain regions where differential activation by erythromycin compared to saline covaries with differences in plasma insulin levels.*** *Color bar represents F-values. pACC, perigenual anterior cingulate cortex; AIC, anterior insular cortex.*

## Supplementary References

1 Janssen, S. *et al.* Bitter taste receptors and alpha-gustducin regulate the secretion of ghrelin with functional effects on food intake and gastric emptying. *Proc Natl Acad Sci U S A* **108**, 2094-2099, doi:10.1073/pnas.1011508108 (2011).

2 Deloose, E., Vos, R., Corsetti, M., Depoortere, I. & Tack, J. Endogenous motilin, but not ghrelin plasma levels fluctuate in accordance with gastric phase III activity of the migrating motor complex in man. *Neurogastroenterol Motil* **27**, 63-71, doi:10.1111/nmo.12470 (2015).

3 Blundell, J. *et al.* Appetite control: methodological aspects of the evaluation of foods. *Obes Rev* **11**, 251-270, doi:10.1111/j.1467-789X.2010.00714.x (2010).

4 Flint, A., Raben, A., Blundell, J. E. & Astrup, A. Reproducibility, power and validity of visual analogue scales in assessment of appetite sensations in single test meal studies. *Int J Obes Relat Metab Disord* **24**, 38-48 (2000).
